# Lower Non-Heme Iron Absorption in Healthy Females from Single Meals with Texturized Fava Bean Protein Compared to Beef and Cod Protein Meals: Two Single-Blinded Randomized Trials

**DOI:** 10.3390/nu14153162

**Published:** 2022-07-30

**Authors:** Inger-Cecilia Mayer Labba, Michael Hoppe, Elisabeth Gramatkovski, Martin Hjellström, Mehdi Abdollahi, Ingrid Undeland, Lena Hulthén, Ann-Sofie Sandberg

**Affiliations:** 1 Food and Nutrition Science, Department of Biology and Biological Engineering, Chalmers University of Technology, 412 58 Gothenburg, Sweden; khozaghi@chalmers.se (M.A.); undeland@chalmers.se (I.U.); ann-sofie.sandberg@chalmers.se (A.-S.S.); 2 Department of Internal Medicine and Clinical Nutrition, Institute of Medicine, Sahlgrenska Academy, University of Gothenburg, 413 45 Gothenburg, Sweden; michael.hoppe@nutrition.gu.se (M.H.); gramati299@gmail.com (E.G.); lena.hulthen@medfak.gu.se (L.H.); 3 Department of Medical Radiation Sciences, Institute of Clinical Sciences, Sahlgrenska Academy, University of Gothenburg, 413 45 Gothenburg, Sweden; martin.hjellstrom@gu.se

**Keywords:** iron, non-heme iron absorption, single meal, fava bean, whole-body counting, fish protein, meat protein, bean protein, protein shift, plant-based

## Abstract

Meat analogs based on plant protein extracts are rising in popularity as meat consumption declines. A dietary shift away from meat, which has a high iron bioavailability, may have a negative effect on the amount of iron absorbed from the diet. Iron absorption from legumes cultivated in regions not suitable for soy production, such as fava bean, has not yet been explored. The aim of this study was to evaluate non-heme iron absorption from a meal with texturized fava bean protein compared to beef and cod protein meals. The study included two single-blinded iron isotope trials in healthy Swedish women of the ages 18–45 years, each of whom served as their own control. The participants were served matched test meals containing beef and fava bean protein (Study 1) or cod and fava bean protein (Study 2) with radiolabeled non-heme iron ^55^Fe and ^59^Fe. The absorption of non-heme iron from test meals was measured by whole-body counting and erythrocyte incorporation. The absorption of non-heme iron, measured as erythrocyte incorporation ratio, from beef protein meal was 4.2 times higher compared to texturized fava bean meal, and absorption from cod protein meal was 2.7 times higher compared to the fava bean meal. The adjusted non-heme iron absorption, normalized to a 40% reference dose uptake, was 9.2% for cod protein meal, 21.7% for beef protein meal, and 4.2% for texturized fava bean meal. A fava bean protein meal has markedly lower iron bioavailability in healthy females compared with a meal of beef or cod protein. Therefore, a dietary shift from meat and fish protein to fava bean protein may increase the risk of iron deficiency.

## 1. Introduction

Due to concerns regarding the climate impact from food consumption, a shift toward a plant-based diet has been pushed as a means to reduce the climate footprint as well as land and water use [1,2,3,4]. Another strong argument commonly used for such a shift is health since a mainly plant-based diet is related to a decreased risk of metabolic diseases if the diet is composed of high-quality foods [5,6]. Yet, a high-quality plant-based diet has also been linked to an increased risk of micronutrient deficiencies, largely due to limited intake of vitamins D and B12, selenium, iodine, and the low bioavailability of minerals [7].

Rather than increasing the intake of health-promoting, high-quality foods, the use of refined products and meat analogs is becoming increasingly common [8]. A common method to achieve a meat-like structure in the production of meat analogs is extrusion cooking, i.e., the texturization of plant protein extracts. The extraction of plant proteins has been shown to accumulate phytate, a potent inhibitor of iron absorption, as well as other antinutritional components, in the protein-rich fraction later used for extrusion cooking [9,10,11,12]. During extrusion cooking, high pressure and high temperature are used to reshape the protein structures. This processing technique has been reported to affect the composition and nutritional quality of plant protein, the magnitude and effect depending on the specific settings during processing [13,14,15]. Extracted and extruded plant protein differs significantly in terms of composition compared to the raw material, and the health implications of such products are largely unknown.

Meat analogs based on extracted plant protein often have a high content of iron [16,17]. However, the uptake of iron from such products is questionable due to the high content of phytate [18,19]. The inhibitory effect of phytates on mineral absorption is explained by the formation of insoluble and indigestible phytate-mineral compounds in the gut [20]. Excessive levels of phytates in the diet can lead to iron deficiency, highlighting the relevance of investigating iron bioavailability rather than iron content [21,22]. Vegetarians have been shown to have lower iron stores and an increased prevalence of iron deficiency compared with non-vegetarians, mainly explained by the lower bioavailability of iron in plant foods [23,24] as plant foods contain no heme iron, no muscle protein stimulating absorption, and in general have a high phytate content. The current prevalence of iron deficiency in women of fertile ages living in Western countries has been estimated to be 10–30% [25,26,27,28]. Women across Western societies are twice as likely as men to follow a plant-based diet [29], which augments the vulnerability of developing low iron status and iron deficiency.

The use of soy for the production of meat analogs is widespread even in countries with no domestic production of soy. Using domestic crops that are possible to cultivate in regions with short growing seasons, such as fava bean [30], is important in regions currently dependent upon the importation of soybean. Fava bean has been highlighted as a crop with upscaling potential in Sweden [31] and has been suggested to be suitable for the substitution of meat [32]. Moreover, soy protein per se has been shown to inhibit non-heme iron absorption [33,34,35]. The inhibitory effect is attributed to the protein fraction conglycinin [36], which is not present in fava bean [37]. Another advantage is that white-flowered cultivars of fava bean contain comparatively low levels of iron-binding polyphenols [38]. Chemical analyses of the composition of whole fava bean have revealed nutritional differences between cultivars and a generally high content of phytate. The Phytate:Fe molar ratios suggest that the bioavailability of iron in whole fava bean seeds is low [39], with the exception of the Sunrise cultivar, which was analyzed in a previous paper by our group [38].

To the best of our knowledge, iron absorption from a meal of extracted and texturized fava bean protein, industrially produced as meat analogs, has not before been measured in human subjects.

The primary aim was to investigate the absorption of non-heme iron from a meal with texturized fava bean protein compared to matched meals with cod and beef protein.

It was hypothesized that non-heme iron absorption from the plant protein meal would be lower than from the animal protein meals.

## 2. Materials and Methods

### 2.1. Subjects

The participants in the study were apparently healthy women aged 18–45. Women of reproductive age were chosen as subjects since this is one of the main risk populations in terms of developing iron deficiency. No iron-containing supplements were allowed two weeks prior to the study, and the donation of blood was not allowed six months prior to the study. Pregnant or lactating women were excluded from the study. Each participant was asked about their current health status and any indications of infection during the weeks prior to the study. Questions regarding infection were repeated at each visit to avoid errors due to infection, as an activated acute-phase reaction influences iron homeostasis and iron absorption [40]. Recruitment was undertaken using flyers set up on university campuses in Gothenburg, Facebook advertisements, and the digital recruitment platform Accindi. Initially, 201 individuals were assessed for eligibility. The sample size (10–15 participants) was determined from the previous literature on similar trials [35,41]. A total of 23 and 22 subjects, respectively, were enrolled in Study Group 1, beef protein vs. fava bean protein meals (beef–fava), and Study Group 2, cod protein vs. fava bean protein meals (cod–fava), respectively (Table 1).

A total of 18 participants were excluded during the study, the main reasons being that they were unable to finish the meal (*n* = 7) and sickness during the study (*n* = 7). Each participant in both study groups started with a texturized fava bean meal, which was perceived as very dry and, therefore, too difficult for some participants to eat. All of the dropouts caused by this reason occurred during the intake of the first test meal. The participants that developed a fever or an infection during the study were excluded, as this would affect iron metabolism. The high rate of sickness in the two trials can be explained by the simultaneously ongoing COVID-19 pandemic. For a detailed description, see the participant flowchart (Supplementary S1 participant flowchart).

### 2.2. Experimental Design: Study 1 (Beef-Fava Bean) and Study 2 (Cod-Fava Bean)

The study included two single-blinded double radio-iron isotope trials in healthy Swedish women aged 18–45 years, where each subject served as her own control. The double radio-iron method enabled the effect of texturized fava bean protein compared to meat or fish protein to be measured separately in the same individual. The two trials were performed from winter to spring 2021–2022.

The subjects were served three potato patties baked with a total of 35 g (dry weight, dw) of protein extract. In Study 1, the subjects were served texturized fava bean protein (B) and beef protein (A). In Study 2, the subjects were served texturized fava bean protein (B) and cod protein (A). The subjects fasted before they were served the meals as breakfast for four consecutive days in the order BBAA. The meals were served with 2 dL of water. No food or drinks were allowed within three hours after the intake of the meal. The subjects were measured by a sensitive whole-body counter (WBC) approximately 2 weeks after the meal intake, according to Skoldborn et al. [42], with modifications described by Fredlund et al. [43]. A fasting blood sample followed by the intake of a reference dose containing ^59^Fe was taken at the Sahlgrenska hospital, Gothenburg, after a WBC measurement. Approximately 2 weeks after the intake of the reference dose, the subjects were again measured by a WBC in order to adjust for individual differences in absorption. The subjects were randomly allocated by computer randomization performed by a colleague not involved in the study. Randomization was restricted to the two study groups (beef–fava or cod–fava).

### 2.3. Preparation and Serving of Test Meals

Each meal was based on a dough consisting of boiled and mashed potato (180 g, after boiling), 38 mL of tap water, 15 mL of rapeseed oil, and 8 mL of mushroom broth (containing water, salt, E621, E631, yeast extract, sugar, maltodextrin, potato starch, mushroom, E202, E412, E415, flavors, and ascorbic acid). The added content of ascorbic acid was 0.8 mg per dough portion. Since ascorbic acid is a heat-sensitive vitamin, the content in the ready meal was considered negligible and to have no impact on the iron absorption from the meals.

To each dough, 35 g dw of protein was added. Due to the differences in protein content between the three dried and milled protein sources, the cod meal consisted of 44 g of protein concentrate (with a protein content of 83.4%, dw), the beef meal consisted of 42 g of protein concentrate (82.9% protein content, dw), and the fava bean meal contained 67 g of protein concentrate (56.3% protein content, dw). The dough was divided into three equally-sized burger-shaped pieces, and these were baked in a conventional oven at 200 °C for 28 min. After baking, the meal was allowed to cool before the three pieces were placed into a microwave-safe container and stored at −18 °C. The containers were marked with a color code for each type of meal; the code was not visible to the participants. The afternoon before the meals were served, the meals were placed in a refrigerator and allowed to thaw until the following morning when the meals were microwave-heated, and the iron isotope was added. The meals were served outside the clinical nutrition test kitchen at the Sahlgrenska Academy, Gothenburg.

### 2.4. Radioiron Labeling of Meals

To determine the amount of iron absorbed from the meals, extrinsic radio-labeled ^59^Fe (A, beef and cod meals) and ^55^Fe (B, texturized fava bean meals) were added as FeCl_3_ in HCl to the surface of the heated meals by pipetting immediately before serving. The isotopes ^55^Fe and ^59^Fe have been used in iron-absorption studies for nearly 80 years since their introduction in 1939 by Hahn et al. [44]. Extrinsic radio-iron labeling techniques rely on the basis of the “pool concept”, which is the isotopic exchange through diffusion between extrinsically-added iron and the native iron in the food. It has been shown that from a single food that has been biosynthetically labeled with iron using an isotope of either ^55^Fe or ^59^Fe and mixed with extrinsically-added iron in the other isotopic form, the absorption of the two isotopes is identical. The absorption of extrinsic and intrinsic isotopes has been shown to remain identical even in meals with different iron bioavailability [45,46], with the exception of foods with a hard outer layer, which inhibits diffusion [47]. In the test meals used in the present study, we expected no interference with the isotopic exchange between the native and extrinsic iron.

### 2.5. Iron Absorption Measurements

The primary outcome measure was the iron absorption from the test meals, which was measured using two different radio-iron tracers. The average radioactivity each subject received was a total of 74 kBq from ^55^Fe and 103.6 kBq from ^59^Fe (34 kBq from the reference dose and 70.0 kBq from the meal).

The iron absorption from the ^59^Fe-labelled beef and cod protein meals was calculated as the detected whole-body radioactivity content at 11–15 days after the meal intake, corrected for physical decay and background radiation, and compared to the measured ^59^Fe radioactivity in the served meal. The whole-body content of ^59^Fe was measured for 10 min per subject by a whole-body counter (WBC) situated in the Sahlgrenska University hospital and consisting of two NaI-detectors (“5 × 4”) located above and below the subject’s thorax region in a stationary lying-on-a-bed geometry [43]. The reference dose, used for the absorption normalization between the individuals, was measured in the WBC, 12–16 days after the intake of the reference dose. For s detailed method description of WBC, see Supplementary Data S2 Detailed method description for whole body counting measurements.

Since the activity of ^55^Fe cannot be detected by a WBC due to a lack of appropriate gamma-ray emissions, the blood sample that was drawn immediately after the first WBC (11–15 days after meal intake) was used to determine the ratio between the two isotopes using a liquid scintillator. The isotope ratio was then used to calculate the ^55^Fe absorption based on the measured ^59^Fe activity from the WBC measurements. The analysis was performed according to the method described by Eakins et al. [48], with slight modifications by Hoppe et al. [49]. Duplicates of whole blood corresponding to 10 mg of Fe were pre-treated and analyzed in a liquid scintillator (Tri-Carb, Packard Instruments, Dallas, TX, USA) to determine the radiation from ^55^Fe and ^59^Fe.

### 2.6. Oral Reference Dose

By relating the absorption from the test meals to the absorption of a reference dose, the variation depending on individual differences in iron absorption capacity, primarily dependent on iron status, can be corrected [50]. Therefore, a reference dose of ferrous ascorbate with added ^59^Fe was administered to the participants. Iron absorption was normalized to a 40% absorption from the reference dose, corresponding to borderline iron-deficient individuals who have not developed anemia, as this is a standard procedure and allows for comparisons between the two trials as well as other iron absorption studies [50]. The reference doses are usually administered to participants two days in a row in order to limit day-to-day variations within the same individual [51,52], but due to the global shortage of ^59^Fe as a consequence of the ongoing COVID-19 pandemic, it was only possible to use one reference dose.

### 2.7. Statistical Analyses

The data analysis was performed with an IBM SPSS version 28 (IBM, New York, NY, USA). To investigate the differences between the groups and to account for the small sample size and skew, a Mann–Whitney U test was used, and *p*-values below 0.05 were considered significant. The absorption quota is presented as the ratio between ^59^Fe/^55^Fe in erythrocytes. The percentage of absorption is presented as the median uptake using combined WBC/erythrocyte data, and for fava bean meal absorption, this was calculated using the combined data from Study 1 and Study 2 as no significant difference in ^55^Fe absorption was found between the two study groups.

### 2.8. Hematological Analysis

On the first day of the study, each participant gave a fasting blood sample at the Sahlgrenska hospital, Gothenburg, by venipuncture prior to the intake of the first meal. The blood was analyzed at an accredited reference laboratory (Clinical Chemistry Laboratory, Sahlgrenska University Hospital, Gothenburg, Sweden) for C-reactive protein (CRP) and iron status: serum iron concentration, total iron binding capacity (TIBC), transferrin saturation (TSAT), serum ferritin, soluble transferrin receptor (sTfR), and hemoglobin. CRP was analyzed to avoid any errors caused by ongoing infection.

### 2.9. Extraction of Beef- and Cod Protein

Fresh, ice-covered Atlantic cod (*Gadus morhua*) filleting co-products, including the head and tail, were provided by Fisk Idag AB and Sjöboden AB (Gothenburg, Sweden). Fresh boneless beef loins were also bought from a local store. Right after arrival, the co-products and beef samples were separately ground using a table-top meat grinder (C/E22 N, Minerva Omega Group, Italy) equipped with a plate with 4.5 mm holes. Then, the mince produced from the co-products and beef samples were packed in polyethylene Ziplock bags and frozen and stored at −80 °C until further usage.

To extract protein from the minced samples, they were subjected to alkaline solubilization and isoelectric precipitation following the main steps reported by Abdollahi and Undeland (2019) [53] with minor modifications. For a detailed description of the method, see Supplementary Data S3 Extraction of beef and cod protein.

### 2.10. Analysis of Food Composition

The three meals were analyzed for their content of Fe and Zn, protein, total fat, and the presence of total phenolics. The meat and cod meals were analyzed for their content of heme iron, and the fava bean meal was analyzed for its content of phytate.

The total iron and zinc contents in the protein extracts were determined in duplicate by atomic absorption spectrometry (200 Series AA System; Agilent, Santa Clara, CA, USA). The digestion of the samples prior to analysis was performed using the microwave technique, as previously described by Fredriksson et al. [54]. The heme-iron content in the cod and meat protein isolates was determined in triplicate by HPLC, according to the protocol developed by Ryter and Tyrell [55]. The protein content was determined by the Dumas principle using a LECO Trumac nitrogen analyzer (LECO Corporation, St. Joseph, MI, USA). The total protein content of the meat was calculated using a nitrogen-to-protein conversion factor of 5.42, fish was 5.58, and fava bean was 5.4, according to Mariotti et al. [56]. The total phenolic content (TPC) was determined in duplicate by the Folin–Ciocalteu method, based on the technique of Howard et al. [57]. Phytate was analyzed as inositol hexaphosphate (InsP_6_) by high-performance ion chromatography (HPIC), according to Carlsson et al. [58]. The lipids were analyzed and extracted with chloroform and methanol (2:1), according to Bligh and Dyer [59], as modified by Lee et al. [60]. The molar ratios Phy:Fe and Phy:Zn in the fava bean protein were calculated to estimate the relative bioavailabilities of iron and zinc, respectively, and to give an indication of the inhibitory effects of phytate on these minerals. A molecular mass for phytate of 660.3 g/mol was used for the calculations.

### 2.11. Ethics

The study protocol was approved by the Ethical Review Board in Gothenburg, Registration Diary number 573-18. The subjects were given comprehensive written and oral information about the aims and procedure prior to study inclusion. A written consent form was signed by the subjects before the study started. The trial was registered at www.clinicaltrials.gov as NCT05392816.

## 3. Results

### 3.1. Chemical Composition of Meals

The protein content was matched between the three test meals (Table 2), while the content of iron and zinc was higher in the fava bean meal compared to the cod and beef meals. The fava bean meal contained a high level of phytate, with a phytate-to-iron molar ratio of 39, and a phytate-to-zinc molar ratio of 51.

### 3.2. Iron Absorption from Meals

The hematological data and age did not significantly differ between the two study groups (Table 3). However, the BMI was significantly higher in the beef–fava study. The average BMI in the beef–fava group was 25.5, which corresponds to overweight. The average BMI in the cod–fava group was 22.6, which corresponds to a normal weight. Large individual differences were found as expected within the two study groups with respect to iron status. The CRP level of the participants was low on the first day of the trial; no exclusions were made based on the blood analysis.

The absorbance quota, analyzed by the erythrocyte-incorporated iron using liquid scintillation, was 2.7 times greater for the cod-protein meal compared to the texturized fava bean meal and 4.2 times greater for the beef-protein meal compared to the texturized fava bean meal.

The absolute measurements of ^59^Fe absorbance from WBC were combined with the relative ^59^Fe/^55^Fe absorbance quota from the erythrocyte incorporation analysis by liquid scintillation in order to calculate the absorbance of ^55^Fe. The beef- and cod-protein meals were labeled with ^59^Fe, while the texturized fava bean protein meals were labeled with ^55^Fe.

**Figure 1:**
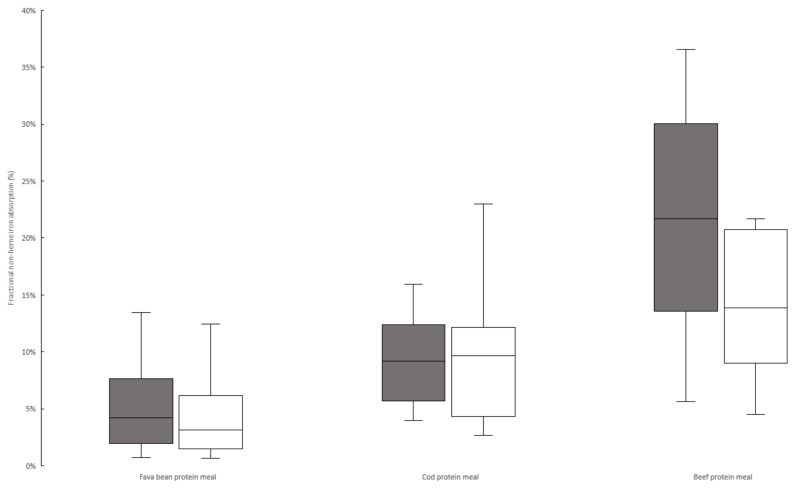
Box plot of adjusted and unadjusted non-heme iron absorption from texturized fava bean meal, cod-protein meal, and beef-protein meal. Absorption of texturized fava protein is based on combined data from Study 1 and Study 2. Grey boxes represent data adjusted to a 40% reference dose absorption; white boxes represent unadjusted data. Adjusted and unadjusted data based on combined data from absorption quota and WBC was significantly (*p* < 0.05) different between each of the three meals, analyzed by Mann–Whitney U test.

As seen in Figure 1, the adjusted median fractional absorption of non-heme iron from the texturized fava bean protein meal was 4.2% (3.2% unadjusted), 9.2% for the cod-protein meal (9.7% unadjusted), and 21.7% for the beef-protein meal (13.9% unadjusted). The beef–fava group had an insignificant trend of lower absorption from the reference dose (Table 3). As we adjusted the absorption based on the reference dose uptake, the correction for this group was higher, which explains the larger difference between the adjusted and unadjusted absorption from the beef-protein meal compared to the cod-protein meal. There was no significant difference in the fava bean meal iron absorption between the two study groups.

According to the Swedish national dietary survey, Riksmaten, Swedish women of fertile ages have a total meat intake of 88 g per day on average [61]. Total meat includes red meat, poultry, offal, blood products, and sausages. According to the Nordic Nutrition Recommendations, the daily need for iron is 2.22 mg per day for women of fertile ages [62]. The contribution to the daily iron need from current meat intake was calculated to be 18% (Table 4). The calculations were based on a non-heme iron absorption of 21.7% from meat, as found in this study, and an assumption of 25% absorption from heme iron, as previously reported by Monsen et al. [63].

## 4. Discussion

Non-heme iron absorption, measured as ^59^Fe/^55^Fe ratio in erythrocytes, from meals containing beef or cod protein was 4.2 and 2.7 times higher, respectively, compared to a meal with texturized fava bean protein. Combined data from WBC and erythrocyte analysis showed that the absorption of non-heme iron was 9.2% from the cod protein meal (9.7% unadjusted), 21.7% from the beef protein meal (13.9% unadjusted), and 4.2% from the texturized fava bean meal (combined data from Study 1 and Study 2. Unadjusted 3.2%). Apart from the non-heme iron, a large contribution to total iron absorbed can be expected from heme iron in the beef and cod proteins. Approximately 40% of the total iron in these animal protein sources was found to be heme iron, from which up to 25% is considered to be absorbed [66]. Based on this assumption, the total amount of iron absorbed was estimated to be 0.62 mg from the beef protein meal (out of 2.7 mg total iron), 0.36 mg from the cod protein meal (out of 2.3 mg total iron), and 0.16 mg from the fava bean meal (out of 3.7 mg total iron). Despite having the highest amount of total iron, the actual iron absorbed from the fava bean meal was significantly lower compared to the beef and cod protein meals. Compared to the daily iron need of women of fertile ages, which is estimated to be 2.22 mg/day (90th percentile) according to the Nordic Nutrition Recommendations [62], the contribution from the fava bean meal was 7.0%. The contribution to the daily need for iron from the beef protein meal was 28.0% and 16.3% from the cod protein meal. A total exchange from current meat intake among Swedish women into texturized fava bean protein was calculated to result in a decrease of 82% of absorbed iron from what was expected from the meat fraction, as the texturized fava bean was calculated to contribute with 3% of the daily iron need (Table 4). Exchanging half of the current total meat intake was calculated to result in a 41% decrease of absorbed iron from what was expected from the meat fraction and a total contribution of 11% to the daily iron need.

Our results highlight the difficulties of covering daily iron needs with plant-protein-extract products as a substitute for beef or cod meals, especially for women of fertile ages as well as growing individuals with high iron needs. This is an important finding as the majority of meat substitutes currently on the market are based on legume protein extracts, with extrusion as the principal technique for building structure [8]. The effects on health and nutrition of a diet composed of such products are currently unknown [67].

Apart from containing heme iron, meat and fish have been demonstrated many times to enhance non-heme iron absorption ascribed to the meat factor, which is thought to be related to the protein, or peptide digestion products, of muscle tissue [68,69,70,71]. Hurrell et al. showed that extracted heme-free beef protein enhances non-heme iron absorption to the same extent as the native muscle tissue [33].

There was a higher mineral content in the fava bean meal compared to the beef and cod protein meals. Since we wanted the meals to reflect actual protein meals, adjustment of iron content was deemed undesirable. The fava bean meal contained a high content of phytate, which reflects an actual meal based on plant protein extract. The meal was not designed to be high in phytate; rather, the extract naturally had a very high content since phytate is accumulated in the protein fraction of legumes [18]. The molar ratio of phytate to iron was 39, which can be considered to reflect a very low theoretical bioavailability of non-heme iron. A maximum ratio of 1, or ideally 0.4, has been recommended [72]. The molar ratio of phytate-to-zinc was 51, which similarly reflects a very low bioavailability of zinc [73]. In the present study, the low theoretical value of non-heme iron absorption was confirmed by the clinical trial results. Reducing phytic acid is an essential means of achieving adequate non-heme iron absorption from plant-based meals [74,75,76]. The total iron content has been reported to be higher among plant-based dietary patterns compared with omnivore dietary patterns [77,78,79,80], while individuals following plant-based diets have been reported to have an increased prevalence of low iron stores and iron deficiency [23,24,81]. Our findings suggest that the content of iron in plant protein should be evaluated in a different way, taking into account the bioavailability, rather than solely total content.

As mentioned, only one reference dose of ^59^Fe was administered to the participants. Day-to-day variations of iron absorption are known to be significant. Therefore, it is most likely that among the participants in the study, day-to-day variations have had an impact on the amount of iron absorbed from the reference dose. Despite this limitation, one reference dose was still used as an attempt to adjust for individual differences and comparisons between the two study groups as well as other trials. The absorption of the reference doses was 39% in the cod–fava study group and 30% in the beef–fava study group. The mean reference dose absorption did not differ significantly between the two study groups (*p* ≥ 0.05), but the adjustment of ^59^Fe in the beef-fava study group was higher due to a trend of lower reference dose absorption in the beef group, which might be explained by day-to-day variations. Since overweight and obesity are related to an impairment of iron absorption [82,83], the beef–fava group may potentially have absorbed less iron from the meals and reference dose compared to the cod–fava group, but no difference could be found in the absorption of ^55^Fe (fava bean meals) between the two groups.

Our results are in line with previous studies on plant foods in single meals [35,74,84,85,86] as well as reports on iron status in long-term interventions [87]. Even though single meal absorption studies tend to exaggerate the inhibitory effects compared to long-term studies [88], our results highlight a need to update recommendations with regard to iron and low-bioavailability meals. Today, most recommendations on iron intake are calculated on a relatively high bioavailability of iron in the diet. For example, Nordic Nutrition Recommendations (NNR) calculate recommended daily intake of iron based on a bioavailability of 15%, which corresponds to a diet containing meat [62]. There are no specific recommendations for low-bioavailability diets, such as vegetarian or vegan diets. As mentioned, the prevalence of iron deficiency among women of fertile ages across western societies today is approximately 25% [25]. This proportion is at risk of increasing as a result of the protein shift, especially for individuals in vulnerable groups such as women of fertile ages, children, and adolescents, due to their high iron requirements. It is of critical importance to include nutrition and bioavailability in policy-making, calculations, and processes concerning the protein shift in order to counteract potential negative health effects of a dietary shift.

## 5. Conclusions

Clearly, the substitution of beef and cod protein with texturized fava bean protein has a serious negative effect on the amount of iron absorbed from a meal; here, for the first time, it was shown when serving subjects these protein sources in the form of protein extracts. There is a need to develop plant-protein products with high iron bioavailability, especially meat substitutes and meals aimed at young women and children. The results also point out a need to update guidelines on dietary iron intake from plant-based food patterns with low iron bioavailability.

## Figures and Tables

**Table 1:** Exclusion and final inclusion of participants.

	Study Group	
	Beef-Fava	Cod-Fava
Subjects inititally enrolled *, *n*	22	23
Subjects excluded, by cause:		
not being able to finish meal, *n*	5	2
Sensitivity to the fava meal/IBS, *n*	-	1
failing to draw blood, *n*	1	-
sickness during study, *n*	3	4
mixed up meals, *n*	-	1
broke fasting, *n*	1	-
Subjects finishing study and included in analysis, *n*	12	15

* Subjects enrolled from a total of 201 individuals assessed for eligibility.

**Table 2:** Composition of test meals.

	Beef Protein Meal	Cod Protein Meal	Texturized Fava Protein Meal
Protein (g)	35	35	35
Phytic acid (mg)	-	-	1692
Iron (mg)	2.7	2.3	3.7
Zinc (mg)	0.49	0.28	3.3
Phy:Fe molar ratio	-	-	38.7
Phy:Zn molar ratio	-	-	50.8
Heme iron			-
Total Phenolic Content (mg GAE/g)	-	-	0.39
Total fat (g/100 g)	8.3	8.4	6.4

Abbreviations: GAE, Gallic Acid Equivalent.

**Table 3:** Subject data for Study Group 1 (beef–fava) and Study Group 2 (cod–fava).

	1. Beef-Fava		2. Cod-Fava		
	Median (25th–75th)		Median (25th–75th)		*p*-Value ^1^
Included subjects (*n*)	12		15		-
Age (yrs)	30.5	(25.3–37.3)	27	(23–36)	NS
Height (cm)	168	(162–172)	168	(163–172)	NS
Weight (kg)	71	(65–80)	63	(59–72)	0.036
BMI (kg/m^2^)	26	(24–28)	23	(21–24)	0.002
S-Iron (umol/L)	18	(15–24)	19	(13–25)	NS
TIBC (umol/L)	69	(62–75)	70	(65–79)	NS
TSAT (%)	27.5	(22–32)	26	(21–34)	NS
Serum ferritin (ug/L)	45	(24.8–71.8)	57	(24–81)	NS
Hemoglobin (g/L)	133	(126–135)	131	(127–135)	NS
CRP (mg/L)	0.84	(0.6–1.9)	0.66	(0.51–2.9)	NS
Absorbance quota between animal protein/fava protein meals ^2^	4.2 *p* = 0.0012	(3.2–6.7)	2.7 *p* = 0.001	(1.8–3.4)	0.013 ^3^
Reference dose absorption (%)	28	(21–33)	37	(27–45)	NS (0.079)
Absorption of ^55^Fe from fava meals (unadjusted/adjusted), (%)	3.8/5.7		3.0/3.2		NS/NS

Hematological data were analyzed on blood drawn on the first day of the trial as a fasting blood sample. Abbreviations: BMI, body mass index, CRP, C-reactive protein; TIBC, total iron binding capacity; TSAT, transferrin saturation. ^1^ Mann–Whitney U test was used to analyze differences between study groups. ^2^ *p*-value presented for absorbance quota within study groups was analyzed based on liquid scintillation data from respective animal protein(^59^Fe)/fava protein(55Fe). ^3^ *p*-value for absorbance quota between Study group 1 and Study group 2.

**Table 4:** Scenario calculations on the effects of absorbed iron when 100%, and 50% of the total meat intake is exchanged to texturized fava bean meals.

	Reference Diet	Scenario 1	Scenario 2	
	Swedish National Diet	100% Exchange	50% Exchange	
	Total Meat (88 g)	Texturized Fava Bean (88 g)	Meat (44 g)	Texturized Fava Bean (44 g)
Protein (g)	19.7	16.6	9.8	8.3
Zinc (mg)	2.8	1.6	1.4	0.8
Total iron (mg)	1.7	1.8	0.8	0.9
Non-heme iron (mg)	1.0	1.8	0.5	0.9
Heme iron ^1^ (mg)	0.67	0	0.3	0
Non-heme iron absorption ^2^	21.7%	4.2%	21.7%	4.2%
Assumed heme iron absorption ^3^	25%	-	25%	-
Calculated iron absorbed (mg)	0.39	0.07	0.19	0.04
			0.23	
Difference in iron absorbed ^4^		−82%	−41%	
Percentage of daily iron need (2.22 mg)	18%	3%	11%	

Contribution of nutrients from the daily meat intake and from the texturized fava bean meal, respectively, was calculated and shown in the table above. Total meat includes meat from beef, pork, lamb, game, horse, poultry, offal, blood products, and sausages. Red meat, offal, and blood products contribute to the diet of Swedish women of fertile ages with 53 g/dag, poultry with 20 g/day, and sausages 15 g/day. Intake according to the population-based Swedish dietary survey Riksmaten (Swedish national diet) [61]. Nutritional composition of total meat intake was calculated based on the Swedish Food Agency food database [64] using the products “beef sirloin steak pan fried”, “chicken meat cooked fried grilled” and “cooked falu sausage baked” as representative products for each meat category, as reported by Riksmaten. ^1^ Heme iron was calculated based on the general assumption that 40% of the total iron in meat consist of heme iron [65]. ^2^ Non-heme iron absorption was calculated based on the adjusted absorption values in the trials performed in this paper. ^3^ Heme iron absorption was assumed to be 25%, according to the previous work by Monsen et al. [63]. ^4^ Compared with the contribution from meat in the reference diet.

## Data Availability

Data described in the manuscript, code book, and analytic code will be made available upon request pending approval.

## Supplementary Materials

The following supporting information can be downloaded at: https://www.mdpi.com/article/10.3390/nu14153162/s1, Supplementary S1: Participant flow chart; Supplementary S2: Whole-body counting method description; Supplementary S3: Detailed method description of beef and cod protein extraction.
