# Supplementary material for: Lower Non-Heme Iron Absorption in Healthy Females from Single Meals with Texturized Fava Bean Protein Compared to Beef and Cod Protein Meals: Two Single-Blinded Randomized Trials
Source: Nutrients. 2022 Jul 30;14(15):3162. doi: 10.3390/nu14153162 (PMC9370477; doi:10.3390/nu14153162)
Supplement: Supplementary file 1 [file nutrients-14-03162-s001.zip › nutrients-1797538-supplementary.pdf]

Supplementary S1. Participant flowchart

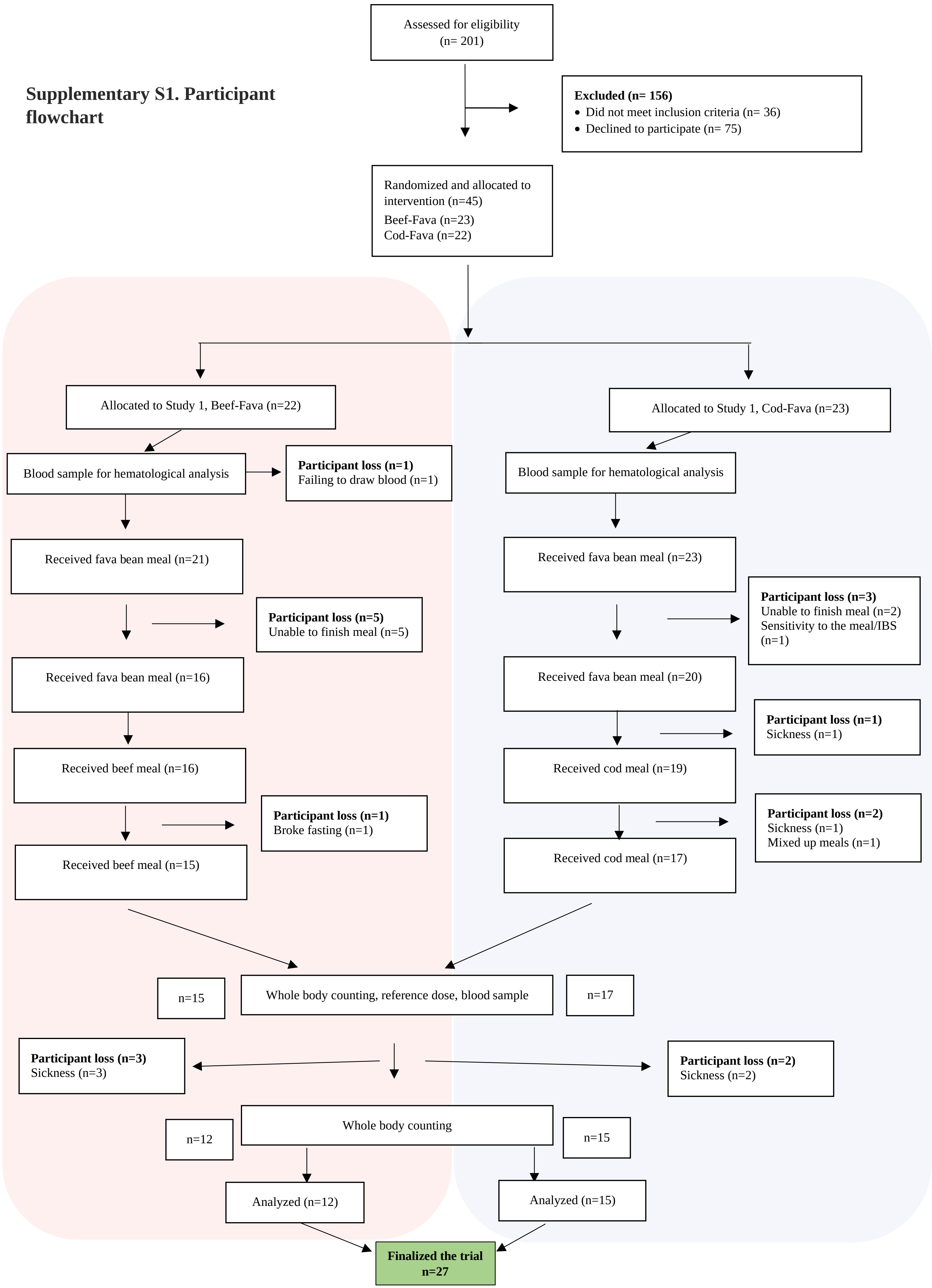

Supplementary S2. Detailed method description for whole body counting measurements

Calibration factors for the ^{59}Fe photopeak of 1099 keV was derived from a heterogenous calibration of ^{60}Co for 6 different body sizes (12-110kg), mimicking the heterogenous distribution of iron in the body [1, 2]. The detection efficiency was assumed to be linear in the energy interval of the main photopeaks of ^{59}Fe and ^{60}Co in the log-log-curve. Body-weight-dependent calibration factors was derived from a curve fit between the 6 available calibration factors and used based on the subject's weight. The background radiation was subtracted in the spectrum analysis for each subject, as the linear interpolation of the continuum on each side of the photopeak [3]. The measured activity was then physical decay corrected individually according to days past since meal intake (11-15 days). Absorption of ^{59}Fe activity from the reference dose was measured and calculated in the same manner. Correction for loss of ^{59}Fe due to the blood sample taken before the reference dose intake and correction for the remaining ^{59}Fe body content from the meal intake was done. The remaining activity of ^{59}Fe from the meal intake were decay corrected to the day of the WBC measurement of the reference dose, and then subtracted from the measured activity of the reference dose.

The ^{59}Fe activity served in each meal was determined by pipetting the same amount that was served into a beaker and then measured in a High Purity Germanium detector (HPGe, 52% relative efficiency). The same procedure was done for the reference dose.

References

1. ICRP, *Occupational Intakes of Radionuclides: Part 2*, I.P. 134, Editor. 2016: Ann. ICRP 45(3/4), 352.
2. ICRP, *Basic Anatomical and Physiological Data for Use in Radiological Protection Reference Values*. 2002: ICRP Publication 89. Ann. ICRP 32(3-4).
3. Knoll, G.F., *Radiation Detection and Measurement*. 2010: Wiley.

Supplementary S3. Extraction of beef and cod protein

A portion of mince from cod head and frame or beef loin was mixed with 6 parts of cold tap water (<10°C) and homogenized with a hand blender for 5 min. Then, the homogenate was adjusted to pH 11.5 using 2M food grade NaOH and incubated at this pH for 10 min. Afterward, the solubilized proteins were separated from non-soluble fractions in the start biomasses using a Russell Finex vibrating sieve (Russell Finex, UK). The pH of the separated protein was then adjusted to pH 5.5 using 2M food grade HCl and incubated for 1 hour at this pH to induce protein coagulation and precipitation. Then, the coagulated proteins were separated from the process water using the Russell Finex vibrating sieve. The recovered proteins were packed in polyethylene Ziplock’s and stored at -80 °C until freeze drying.
